# Genome-wide association study of image-based emphysema scoring in the Swedish CArdioPulmonary bioImage Study (SCAPIS) suggests two new risk loci in smokers

**DOI:** 10.1186/s12931-026-03853-6

**Published:** 2026-08-08

**Authors:** Fredrik Nyberg, Per Lundmark, Anders Blomberg, Koen Dekkers, Arne Egesten, Jonas Eriksson Ström, Bruna Gigante, Anders Gummesson, Cecilia Gunnarsson, Christer Janson, Andrei Malinovschi, Anna-Carin Olin, Marju Orho-Melander, Hans Lennart Persson, Ida Pesonen, Magnus Sköld, Stefan Söderberg, Hanan Tanash, Lowie E. G. W. Vanfleteren, Tove Fall

**Affiliations:** 1 School of Public Health and Community Medicine, Institute of Medicine, Sahlgrenska Academy, University of Gothenburg, Gothenburg, Sweden; 2 Department of Medical Sciences, Molecular Epidemiology, Uppsala University, Uppsala, Sweden; 3 Department of Public Health and Clinical Medicine, Umeå University, Umeå, Sweden; 4 Department of Clinical Sciences Lund, Respiratory Medicine, Allergology, & Palliative Medicine, Lund University and Skåne University Hospital, Lund, Sweden; 5 Department of Medicine, Division of Cardiology, Karolinska Institutet, Stockholm, Sweden; 6 Department of Cardiology, Karolinska University Hospital, Stockholm, Sweden; 7 Department of Molecular and Clinical Medicine, Institute of Medicine, Sahlgrenska Academy, University of Gothenburg, Gothenburg, Sweden; 8 Department of Clinical Genetics and Genomics, Sahlgrenska University Hospital, Region Västra Götaland, Gothenburg, Sweden; 9 Department of Clinical Genetics, and Department of Biomedical and Clinical Sciences, Linköping University, Linköping, Sweden; 10 Centre for Rare Diseases in Southeast Region of Sweden, Linköping University, Linköping, Sweden; 11 Department of Medical Sciences, Respiratory, Allergy and Sleep Research, Uppsala University, Uppsala, Sweden; 12 Department of Medical Sciences, Clinical Physiology, Uppsala University, Uppsala, Sweden; 13 Department of Occupational and Environmental Medicine, School of Public Health and Community Medicine, Sahlgrenska Academy, University of Gothenburg, Gothenburg, Sweden; 14 Department of Clinical Sciences Malmö, Lund University, Malmö, Sweden; 15 Department of Health, Medicine and Caring Sciences, Linköping University, Linköping, Sweden; 16 Department of Respiratory Medicine in Linköping, Linköping University, Linköping, Sweden; 17 Department of Respiratory Medicine and Allergy, Karolinska University Hospital, Stockholm, Sweden; 18 Department of Medicine Solna and Center for Molecular Medicine, Division of Immunology and Respiratory Medicine, Karolinska Institutet, Stockholm, Sweden; 19 Department of Medicine, Skåne University Hospital, Lund University, Lund, Sweden; 20 Department of Internal Medicine and Clinical Nutrition, COPD Center, Sahlgrenska Academy, University of Gothenburg, Gothenburg, Sweden; 21 Science for Life Laboratory, Uppsala University, Uppsala, Sweden

**Keywords:** Computed tomography, COPD, Emphysema, Genetics, Population-based

## Abstract

**Background:**

Despite the high prevalence and clinical significance of emphysema, few genetic risk loci have been consistently replicated. We conducted a genome-wide association study (GWAS) of CT-based emphysema, with a particular focus on non-smoking-related genetic determinants.

**Methods:**

We analyzed 25,639 individuals of European ancestry from the SCAPIS national cohort, aged 50–65 years, of which 51% were never-smokers. Emphysema was assessed through semi-quantitative visual scoring of CT scans. GWAS was performed in the whole sample and stratified on smoking status. We also examined the association of previously reported emphysema- and lung function-related variants with emphysema in our dataset.

**Results:**

Emphysema criteria were fulfilled for 1,479 participants (5.6%), with higher prevalence among current (*N* = 576, 18.2%) and former smokers (*N* = 612, 6.5%) compared to never-smokers (*N* = 263, 2.0%). We identified three independent genetic loci for emphysema in smokers and no signals in never-smokers. The strongest signal was observed in the well-established nicotinic acetylcholine receptor cluster *(CHRNA5-A3-B4)* locus on chromosome 15. Additionally, we discovered novel associations near the *dysferlin (DYSF) gene* on chromosome 2 and in an intergenic region on chromosome 3. By assessing previously lung phenotype-associated variants we also found evidence supporting association with emphysema in smokers for variants in the *EFEMP1/MIR217HG/PNPT1* locus on chromosome 2, previously linked to reduced FEV_1_/FVC ratio.

**Conclusion:**

This study, based on the largest unselected population sample to date, provides novel insights into the genetic architecture of emphysema. However, no signals were detected in never-smokers despite the large sample-size, likely due to the low prevalence of emphysema in that group. The proposed genetic risk loci require external replication.

**Supplementary Information:**

The online version contains supplementary material available at 10.1186/s12931-026-03853-6.

## Introduction

Emphysema represents a key characteristic of chronic obstructive pulmonary disease (COPD), a disease projected to affect 600 million people worldwide by 2050, especially among women and in low- and middle-income countries [1]. Despite the increased recognition of emphysema through imaging in COPD diagnosis [2], as well as emerging specific emphysema-related treatments, genetic determinants underlying emphysema-specific phenotypes, particularly in population-based samples, remain incompletely understood.

One hallmark of emphysema and COPD is reduced lung function. Large meta-analyses of genome-wide association studies (GWAS) have identified > 1,000 loci associated with lung function [3] and > 80 loci associated with COPD [4]. A deeper understanding of the genetic architecture of emphysema could shed light on disease mechanisms, improve risk stratification, and identify novel targets for prevention and therapy. Previous studies on emphysema phenotypes have been limited in size, with somewhat discordant results. An early GWAS of 7,914 individuals in the US Multi-Ethnic Study of Atherosclerosis (MESA) Lung/SNP Health Association Resource (SHARe), for two CT-based emphysema phenotypes, identified and replicated two genome-wide significant loci, *SNRPF* (chromosome 12) and *PPT2/AGER*(chromosome 6), both previously implicated in lung function [5]. Concurrently, the COPDGene study performed GWAS in 9,614 subjects with ≥ 10 pack-years smoking history for five emphysema patterns and reported two novel loci (*MYO1A* and *VMA8)* and five loci (*HHIP, CHRNA5-A3-B4, CYP2A6/ADCK*, *TGFB2*, and *MMP12*) previously linked to COPD [6]. A subsequent GWAS of CT-based emphysema distribution in 11,532 smokers from the COPDGene, ECLIPSE, and GenKOLS studies identified two associations at known COPD loci (*HHIP* and *CHRNA5-A3-B4* at 15q25) and three at novel loci (*SOWAHB*, *TRAPPC9,* and *KIAA1462*) [7]. In an extended analysis of the COPDGene, ECLIPSE, GenKOLS and NETT studies, two additional new loci at SERPINA10 and DLC1 were identified [8]. Another important phenotype in this context is the diffusing capacity of the lungs for carbon monoxide (DLCO). The first population-based GWAS of DLCO from the Rotterdam Study and Framingham Heart Study, identified a functional variant in *ADGRG6*associated with DLCO/Va (DLCO divided by alveolar volume) [9]. A subsequent study identified three additional loci and 12 suggestive loci for a similar DLCO phenotype [10].

Notwithstanding these advances, most genetic studies of emphysema have focused on selected populations of smokers or individuals with COPD, limiting our understanding of emphysema genetics in the general population and in relation to smoking history. Therefore, we aimed to identify genetic risk factors for emphysema—particularly those independent of smoking—using semi-quantitative visual scoring of CT scans in a large, unselected population with diverse smoking habits. We also assessed genetic variants previously associated with emphysema, lung function and COPD for association with emphysema in our data.

## Methods

### Study sample

The Swedish CArdioPulmonary bioImage Study (SCAPIS) (http://scapis.org/) is a major collaborative national project in Sweden to study mortality and morbidity from cardiovascular disease, pulmonary disease and related metabolic disorders [11]. The study was conducted according to the Declaration of Helsinki and with relevant ethical approvals. All study participants provided informed written consent to participate before enrolment in the study. From 2013 to 2018, 30,154 subjects aged 50–65 years were recruited based on a random sample of the population in six areas around six Swedish university hospitals. All participants were studied with an ambitious core program consisting of an extensive questionnaire assessment including smoking habits, at least two visits to the test center, imaging by computed tomography (CT) of the heart, lungs and fat depots (with standardized sequential image acquisition workflow), biochemistry, anthropometry, electrocardiography, blood pressure measurement, 7-day accelerometer, and lung function tests.

### Outcome definitions

Emphysema classification was based on CT imaging with a dedicated dual-source scanner and a Stellar Detector (Somatom Definition Flash, Siemens Medical Solutions) (details in Supplementary Methods). Images were interpreted and recorded in an electronic case report form by each radiology department. Consensus meetings were held pre-study for interpretation consistency. Radiologists were blinded to participant characteristics but could review previous investigations while making CT interpretations.

Three regions in each lung were reviewed: the upper zone above the carina; the middle zone between the carina and inferior pulmonary vein; and the lower zone below the inferior pulmonary vein, using a syngo.via (Siemens Healthineers) thin-slice workstation. Each region was assessed for presence of emphysema, with emphysema graded as none, mild (1% to 25%), moderate (> 25%—50%), or severe (> 50%) [12].

In this study, the emphysema score in each region was coded 0–3, and the sum for all six regions constituted a semiquantitative sum score of 0–18 [12]. We also assessed emphysema as absent (sum score 0) vs present (1–18) in a binary analysis.

### Genotyping and imputation

Of the 30,154 individuals in the SCAPIS cohort, blood samples from 29,433 with consent for genetic analysis were submitted for DNA extraction at Karolinska Institutet biobank (ki.se/en/research/ki-biobank). Samples were genotyped in 10 batches using a customized Illumina GSA-MDv3 genotyping array at the SNP&SEQ Technology Platform in Uppsala, part of the National Genomics Infrastructure Sweden and Science for Life Laboratory. Genotypes were called with the Illumina GenomeStudio 2.0.3 software, with genotype clusters defined in the first batch and then applied to subsequent batches for consistent cluster limits. Genotyping batches were monitored for genotype/phenotype sex discordance, marker and sample missingness, heterozygosity rates, and batch effects related to the DNA plate. Two samples failed genotyping due to low DNA concentration, resulting in a total autosomal genotyping rate of 0.998 and a dataset with 29,431 genotyped samples and 726,358 markers. The Sanger imputation service server was used for genotype imputation using the HRC r1.1 reference panel [13] (details in Supplementary Methods). Of the 29,425 individuals remaining with high-quality imputed data, we then excluded individuals with sex aneuploidy (*N* = 11), non-European ancestry (*N* = 2,048), heterozygosity deviations > ± 3 SD (*N* = 239), and genotype call rates < 99% (*N* = 120), missing high-quality imaging (*N* = 661) and missing data on smoking (*N* = 707), leaving 25,639 individuals for GWAS analysis (Supplementary Fig. 1, Supplementary Methods).

### GWAS analysis and covariates

Our analysis was performed using an ordinal outcome (1–18) and a binary outcome (yes/no). These were performed in the following five strata, resulting in ten GWASes in total:

1. all subjects (*N* = 25,639)
2. never smokers (*N* = 13,020)
3. current smokers (*N* = 3,161)
4. former smokers (*N* = 9,458)
5. ever smokers (combination of current and former; *N* = 12,619)

Genetic exposure was modelled using an additive genetic model, with allele dosages representing the observed (0, 1 or 2) or expected (continuous 0–2) number of effect alleles based on genotype probabilities from the imputation procedure. Covariates included age, age^2^, sex, site and the 10 first principal components of the genotype data. We also included smoking status (current, former, never smoker), pack-years smoked and pipe/cigar smoking status (yes/no) as covariates, to reduce genetic risk related to smoking from the analysis. Smoking status was only included in the full samples and in the ever-smoker analysis. In the never-smoker analysis, we did not include pack-years and pipe/cigar smoking status as it was zero for all observations.

GWAS was performed for the binary outcome using the REGENIE software v 3.3 [14], a modern GWAS software that accounts for population structure and cryptic relatedness and supports binary and quantitative phenotypes and covariates. A dataset for the REGENIE step 1 analysis was created from the directly genotyped dataset filtered for sample and variant missingness > 1%, HWE p < 1e-15, minor allele frequency (MAF) < 1% and general SCAPIS sample QC: sex aneuploidy, non-European descent and heterozygosity outside 3 SD of the mean in European samples. This resulted in a genotype set of 482,907 directly genotyped quality filtered variants. REGENIE step 1 analysis was run with parameters –bt, –bsize 1000 and –lowmem. REGENIE step 2 (association analysis) included imputed markers filtered for info score > 0.7 and MAF > 1%. In addition, due to sparse data, we further applied a Firth correction to p-values < 0.01 in all analyses of binary outcomes using the REGENIE –firth and –pThresh 0.01 arguments [15]. The ordinal outcome was assessed using the POLMM implementation [16] in the GRAB R-package v0.0.3.6. This package supports a mixed model approach to analysis of ordinal phenotypes while dealing with cryptic relations and population stratification. A dense genetic relationship matrix (GRM) was used when fitting the null model with method = POLMM and traitType = ordinal. The GRAB.Marker method was then used for single marker tests versus an ordinal phenotype.

The threshold for declared association was set at the customary GWAS level *p* < 5 × 10^–8^. R v4.4.2 and the topr package v2.0.2 were used to plot Manhattan plots (“manhattan()”) and QQ plots (“qqtopr()”). For regional association plots the package locuszoomr v0.3.8 was used (“locus_ggplot()”).

Population stratification was minimized by restricting analyses to individuals of European ancestry and by adjusting for the first 10 genetic principal components. Mixed-model methods implemented in REGENIE and POLMM further accounted for relatedness and residual population structure; any potential residual inflation was assessed using quantile–quantile (Q-Q) plots and the genomic inflation (lambda) factor.

### Definition of significant independent SNPs and loci

To identify independent genome-wide significant SNPs within loci, we applied a stepwise selection procedure implemented in GCTA-COJO v1.94.1 [17, 18]. This method accounts for linkage disequilibrium (LD) by conditioning on other associated variants within a locus to identify independently associated SNPs. LD was estimated from the study data. GCTA-COJO stepwise selection was performed using –cojo-slct with a genome-wide significance threshold of 5e-8, a window size of 10,000 kb, and a collinearity threshold of r^2^ ≤ 0.9. This procedure was used to identify independent signals within each locus. All reported association statistics (beta, SE, p-values) are derived from the primary GWAS unless otherwise specified.

### Post-GWAS lookups

We queried the Open Targets Platform [19] version 26.03 for information on associated SNPs. This platform integrates evidence linking genetic variants to genes, diseases, and potential therapeutic targets, including large-scale GWAS, molecular QTL data, functional genomics, clinical evidence, and literature-derived associations, covering ~ 79,000 targets, 47,000 diseases, and over 34 million evidence items. For the closest gene to each SNP and potential functional consequences, we used the Ensembl v115, selecting one consequence per gene and considering upstream and downstream distances of up to 1,000,000 bp.

### Analysis of previously associated variants for emphysema and related traits for association with emphysema in SCAPIS

We performed a search in the GWAS Catalog and identified three relevant studies that reported results in Europeans for emphysema phenotypes [5–7] and two for DLCO [9, 10]. We also included results from the largest meta-analyses on lung function [4] and COPD [3]. We extracted summary statistics for all SNPs with *p* < 5 × 10⁻⁸ from original publications or from GWAS Catalog. If the study was multi-ancestry, we used results from European ancestry. Alleles were aligned to the trait-increasing allele for consistency. Genome build 38 coordinates in the GWAS catalog were converted to build 37 via the R-package rtracklayer v1.64.0 and the function liftOver() using the hg38ToHg19.over.chain (https://hgdownload.soe.ucsc.edu/goldenPath/hg38/liftOver/). We then extracted results from the present GWAS for all smoking strata. Confirmation of an association was considered present with a Bonferroni-corrected *p* < 0.05 for the 1018 unique SNPs from the GWAS catalog tested in our SCAPIS dataset analysis (equivalent to nominal *p* < 4.91 × 10^–5^). When relevant, LD was assessed using LDpair [20].

## Results

We included 25,639 individuals with visually scored emphysema and high-quality genotype data, and complete covariate information (age, sex, site, genetic principal components, smoking status [current, former, never smoker], pack-years smoked and pipe/cigar smoking status), in the analyses. The proportion of individuals with scored emphysema was similar across most of the 6 sites but substantially lower in the Uppsala site (Table 1). This site also had the highest proportion of never smokers and lowest proportion of current smokers. Age was slightly higher among individuals with emphysema and among former smokers. Overall, 1479 individuals (5.6%) had CT findings of emphysema, with a very skewed distribution of emphysema scores (mean 4.1, range 1–18). As expected, emphysema was more common among current smokers (18.2%) and former smokers (6.5%), and less among never smokers (2.0%). COPD and lung function followed a similar pattern. Further characteristics are provided in Supplementary Table 1. We performed GWAS for emphysema coded as binary and ordinal, respectively, in the following five strata: non-stratified, current smokers, former smokers, ever smokers and never smokers. We found no genetic associations in the non-stratified, never smoker and the former smoker analyses.

**Table 1:** Key descriptive characteristics for adults 50–65 years old of European ancestry from the Swedish SCAPIS cohort 2013–2018 with GWAS, emphysema and smoking data available, overall and by emphysema and smoking status

**Characteristics**	**Total**	**Emphysema**		**Smoking**		
		**YES**	**NO**	**NEVER**	**FORMER**	**CURRENT**
	***N* = 25,639**	***N* = 1451**	***N* = 24,188**	***N* = 13,020**	***N* = 9458**	***N* = 3161**
**Study site**						
Göteborg	5148 (20.1%)	297 (20.5%)	4851 (20.1%)	2391 (18.4%)	2016 (21.3%)	741 (23.4%)
Linköping	4613 (18%)	293 (20.2%)	4320 (17.9%)	2665 (20.5%)	1493 (15.8%)	455 (14.4%)
Malmö	5196 (20.3%)	348 (24%)	4848 (20%)	2208 (17%)	2112 (22.3%)	876 (27.7%)
Stockholm	4305 (16.8%)	284 (19.6%)	4021 (16.6%)	2015 (15.5%)	1773 (18.7%)	517 (16.4%)
Umeå	2161 (8.4%)	142 (9.8%)	2019 (8.3%)	1259 (9.7%)	710 (7.5%)	192 (6.1%)
Uppsala	4216 (16.4%)	87 (6%)	4129 (17.1%)	2482 (19.1%)	1354 (14.3%)	380 (12%)
**Age (years)**						
Mean (SD)	57.6 (4.36)	58.7 (4.31)	57.5 (4.35)	57 (4.34)	58.4 (4.29)	57.4 (4.25)
Median (Min, Max)	57.5 (50.1,65.8)	59.2 (50.1,65.8)	57.4 (50.1,65.5)	56.7 (50.1,65.5)	58.8 (50.1,65.8)	57.3 (50.1,65.4)
**Sex**						
Male	12,435 (48.5%)	757 (52.2%)	11,678 (48.3%)	6797 (52.2%)	4142 (43.8%)	1496 (47.3%)
Female	13,204 (51.5%)	694 (47.8%)	12,510 (51.7%)	6223 (47.8%)	5316 (56.2%)	1665 (52.7%)
**Emphysema**						
Yes	1451 (5.7%)	1451 (100%)	0 (0%)	263 (2%)	612 (6.5%)	576 (18.2%)
No	24,188 (94.3%)	0 (0%)	24,188 (100%)	12,757 (98%)	8846 (93.5%)	2585 (81.8%)
**Emphysema score 0–18**						
Mean (SD)	0.23 (1.2)	4.08 (3.14)	0 (0)	0.066 (0.572)	0.26 (1.29)	0.81 (2.22)
Median (Min, Max)	0 (0,18.0)	3 (1.00,18.0)	0 (0,0)	0 (0,14.0)	0 (0,18.0)	0 (0,16.0)
**COPD**						
Yes	444 (1.7%)	145 (10.0%)	299 (1.2%)	50 (0.4%)	233 (2.5%)	161 (5.1%)
No	25,186 (98.2%)	1306 (90.0%)	23,880 (98.7%)	12,966 (99.6%)	9222 (97.5%)	2998 (94.8%)
Missing	9 (0.0%)	0 (0%)	9 (0.0%)	4 (0.0%)	3 (0.0%)	2 (0.1%)
**Pack-years cig**						
Mean (SD)	7.68 (12)	21.5 (16.9)	6.85 (11.1)	0 (0)	13.1 (11.5)	23.2 (14.4)
Median (Min, Max)	0 (0, 102)	21 (0, 102)	0 (0, 98.0)	0 (0, 0)	10 (0, 100)	22 (0, 102)
**FEV1**						
Mean (SD)	3.29 (0.764)	3.04 (0.843)	3.31 (0.756)	3.41 (0.762)	3.20 (0.735)	3.1 (0.781)
Median (Min, Max)	3.21 (0.590, 6.89)	2.98 (0.590, 6.04)	3.22 (0.820, 6.89)	3.34 (0.820, 6.89)	3.10 (0.710, 6.25)	3.01 (0.590, 6.27)
Missing	194 (0.8%)	6 (0.4%)	188 (0.8%)	109 (0.8%)	59 (0.6%)	26 (0.8%)
**FVC**						
Mean (SD)	4.25 (0.980)	4.25 (1.04)	4.24 (0.976)	4.34 (0.998)	4.14 (0.938)	4.15 (0.987)
Median (Min, Max)	4.13 (1.17, 8.68)	4.16 (1.29, 8.19)	4.13 (1.17, 8.68)	4.25 (1.17, 8.68)	4.00 (1.48, 8.19)	4.01 (1.29, 8.19)
Missing	197 (0.8%)	6 (0.4%)	191 (0.8%)	114 (0.9%)	58 (0.6%)	25 (0.8%)
**FEV1/FVC**						
Mean (SD)	0.778 (0.064)	0.715 (0.098)	0.782 (0.059)	0.788 (0.056)	0.775 (0.063)	0.747 (0.082)
Median (Min, Max)	0.79 (0.270, 1.00)	0.73 (0.270, 0.990)	0.79 (0.320, 1.00)	0.79 (0.320, 1.00)	0.78 (0.270, 1.00)	0.76 (0.270, 0.990)
Missing	199 (0.8%)	7 (0.5%)	192 (0.8%)	114 (0.9%)	59 (0.6%)	26 (0.8%)

N (%) unless otherwise indicated

*COPD* Chronic obstructive pulmonary disease, *FEV1* Forced Expiratory Volume in 1 s, *FEV1/FEV* Ratio of FEV1 to FVC, *FVC* Forced Vital Capacity, *SD* Standard deviation

### Current smokers

**Fig. 1:**
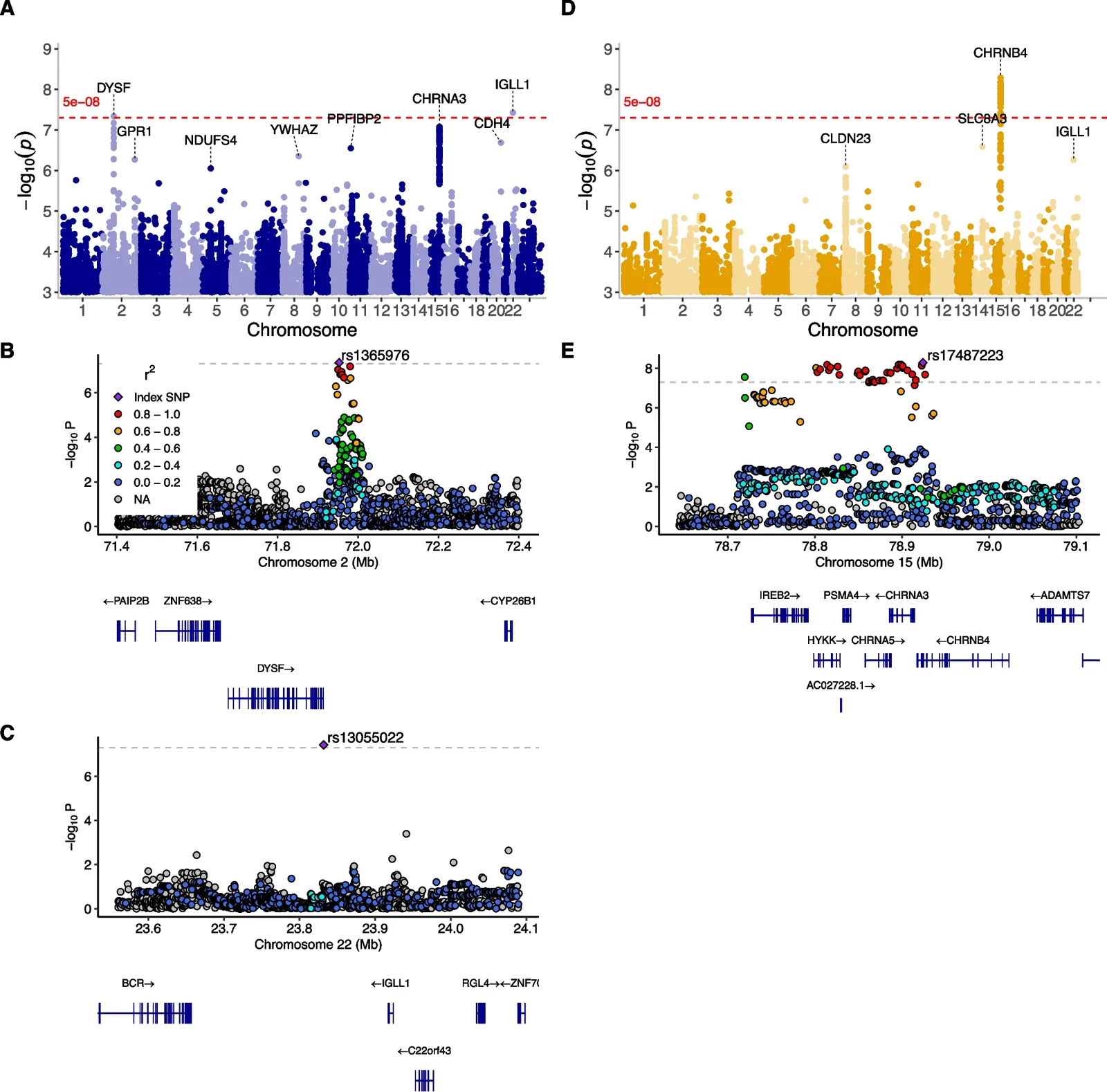
Manhattan plots and regional plots (for signals with *p* < 5 × 10^–8^) for the main analyses in current smokers (*N* = 3,161) for visual scoring of emphysema as a binary variable (A-C) and ordinal variable (D-E), in adults 50–65 years old of European ancestry from the Swedish SCAPIS cohort

Among current smokers (*N* = 3,161), we found associations in three loci (Fig. 1), where one was identified in the ordinal analysis and the other two in the binary outcome analysis: 1) lead variant rs17487223 in the nicotinic acetylcholine receptor cluster (*CHRNA5-A3-B4)* at chromosome 15 *2)* lead variant rs1365976 downstream the *dysferlin (DYSF)* gene on chromosome 2 and 3) lead variant rs1305502 in an intergenic region on chromosome 22 (Table 2, Fig. 1, Supplementary Table 2). Quantile–quantile plots were inspected, with genomic inflation of 1.00 and 1.10, for ordinal and binary traits, respectively (Supplementary Fig. 2). GCTA-COJO analysis did not provide evidence for multiple independent signals within these loci (Supplementary Table 3).

**Table 2:** Results from GWAS analyses for adults 50–65 years old of European ancestry from the Swedish SCAPIS cohort 2013–2018, genome-wide significant SNPs. Analyses in different smoking subsets, with key parameters from the GWAS regression analysis

Population	Model	N	SNP	Chr:Position	EA/OA	EAF	Info score	Beta	SE	P-value	Open Targets evidence*	Closest protein coding genes**
Current smokers	binary	3161	rs1365976	2:71,953,014	A/T	0.53	0.94	−0.42	0.08	4.6 × 10^–8^	QTL: none; GWAS: body weight; Enhancer: none	DYSF (downstream), ZNF638 (downstream), CYP26B1 (downstream)
	binary	3161	rs13055022	22:23,831,823	C/T	0.19	0.73	0.6	0.11	3.7 × 10^–8^	QTL: none; GWAS: none; Enhancer: none	IGLL1 (downstream), DRICH1 (downstream), BCR (downstream)
	ordinal	3161	rs17487223	15:78,923,987	T/C	0.37	0.99	0.41	0.07	5.2 × 10^–9^	QTL: CHRNA5, CHRNA3, PSMA4; GWAS: 84 traits, largely respiratory traits including malignancies; Enhancer: CHRNA3, CHRNB4	CHRNB4 (intron), CHRNA3 (upstream), CHRNA5 (downstream)
Ever smokers	ordinal	12,619						0.26	0.05	2.3 × 10^–8^		
	binary	12,619	rs13074352	3:166,347,433	G/C	0.16	0.99	0.35	0.06	4.6 × 10^–8^	QTL: none; GWAS: none; Enhancer: none	ZBBX (downstream), BCHE (upstream). SERPINI2 (downstream)

*Chr* chromosome, *BCHE* butyrylcholinesterase, *CHRNA3* cholinergic receptor nicotinic alpha 3 subunit, *CHRNB4* cholinergic receptor nicotinic beta 4 subunit, *CHRNA5* cholinergic receptor nicotinic alpha 5 subunit, *CYP26B1* cytochrome P450 family 26 subfamily B member 1, *DRICH1* aspartate rich 1, *DYSF* dysferlin gene, *EA* effect allele, *EAF* effect allele frequency, *IGLL1* immunoglobulin lambda-like polypeptide 1 gene, *info score* information score: A value ranging from 0 to 1 reflecting the quality of imputation for the SNP; OA: other allele; PSMA4: proteasome 20S subunit alpha 4; SE: standard error; SERPINI2: serpin family I member 2; SNP: single nucleotide polymorphism; ZBBX: zinc finger B-box domain containing; ZNF638: zinc finger protein 638

^*^Based on Open Targets Platform 26.03. QTL: Quantitative trait locus (*p* < 5 × 10^–8^). GWAS: 95% GWAS credible set (*p* < 5 × 10^–8^), Enhancer: ENCODE rE2G

^**^Based on Ensembl v115

**Fig.2:**
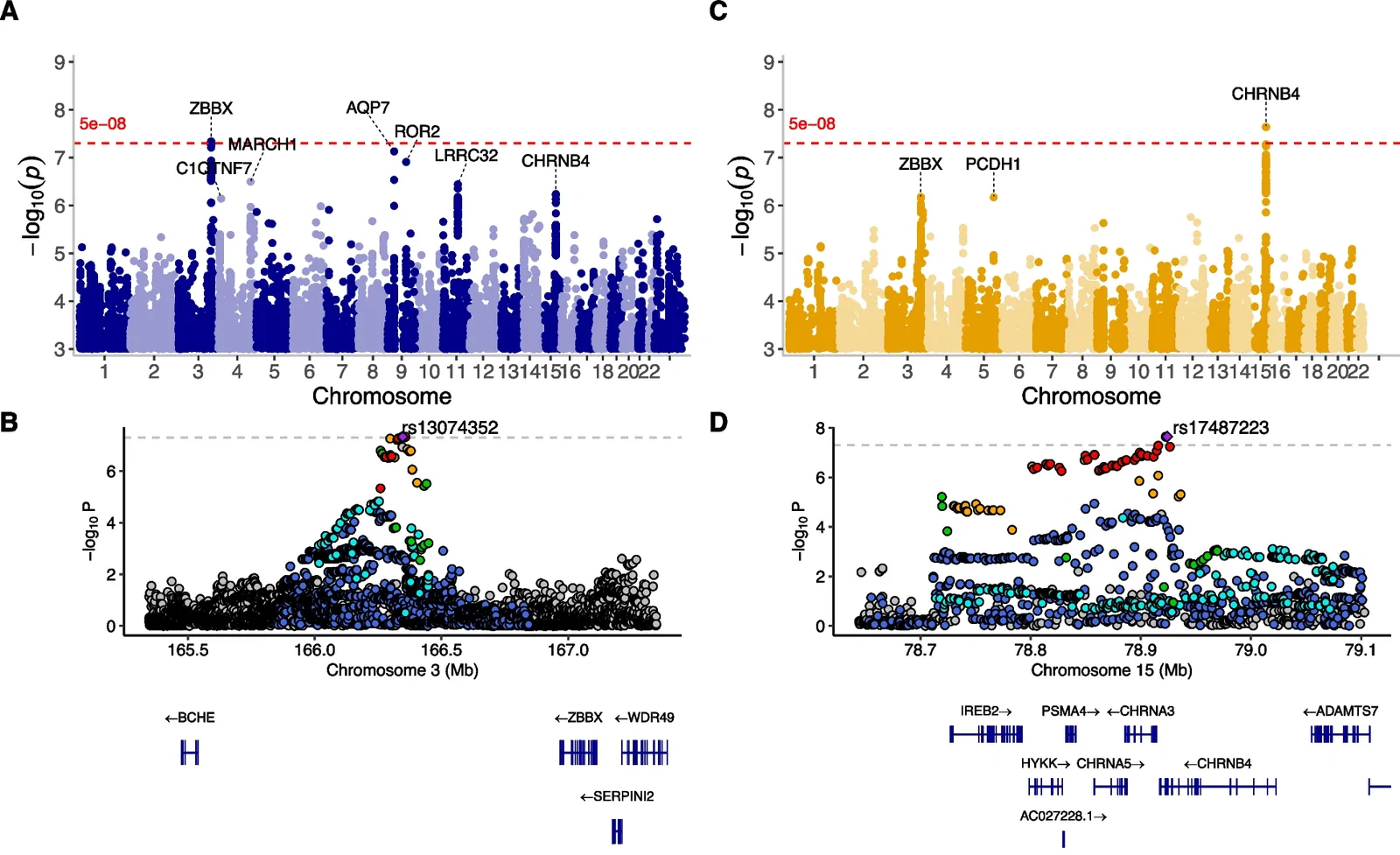
Manhattan plots and regional plots (for signals with *p* < 5 × 10^–8^) for the main analyses in ever smokers (*N* = 12,619) for visual scoring of emphysema as a binary variable (A-B) and ordinal variable (C-D), in adults 50–65 years old of European ancestry from the Swedish SCAPIS cohort

We queried public databases for information on associated SNPs through the OpenTargets Platform. We found that the first locus, *CHRNA5-A3-B4*, is reported to be associated with respiratory and smoking phenotypes in a large number of previous GWAS (Table 2, Fig. 2, Supplementary Table 2, 4–5). The locus is linked to the expression of *CHRNA3, CHRNA5* and *PSMA4.* The second locus (lead variant rs1365976) is previously linked to body weight. There were no findings for the third locus in these public databases. Moreover, regional association plots revealed that the association was only found for a single imputed SNP (Fig. 1C) with relatively low imputation quality (info score 0.72). We hence deem the association of rs1305502 with emphysema unreliable.

### Ever smokers

In the ever-smoker (current and former combined) analysis (*N* = 12,619, Table 2, Fig. 2), the ordinal analysis identified the same associated locus on chromosome 15 as in the current smokers only (lead variant rs17487223). Additionally, in the binary outcome analysis, we found an association in an intergenic region downstream of the *zinc finger B box domain containing (ZBBX)* gene on chromosome 3, with lead variant rs13074352. GCTA-COJO analysis did not provide evidence for multiple independent signals within these loci (Supplementary Table 3). There were no findings for the locus at chromosome 3 in the public databases. Quantile–quantile plots were inspected, with genomic inflation of 1.02 and 1.09, for ordinal and binary traits, respectively (Supplementary Fig. 2).

### Confirmation of variants previously associated with pulmonary phenotypes

We assessed 1018 unique SNPs previously associated at genome-wide significance with relevant lung-related phenotypes, to see whether they were associated with emphysema phenotypes in SCAPIS. Of these, 23 SNPs were previously associated with emphysema imaging pattern, 4 SNPs with diffusing capacity, 251 SNPs with forced vital capacity (FVC), 223 SNPs with forced expiratory volume in one second (FEV₁), 406 SNPs with the FEV₁/FVC ratio, 99 SNPs with COPD and 140 SNPs with peak expiratory flow (Supplementary Table 6). Of the 1018 SNPs, 11 showed Bonferroni-corrected association with emphysema phenotypes in SCAPIS (Table 3, nominal *p* < 4.9 × 10^–5^).

**Table 3:** Association with visually scored emphysema from CT, in adults 50–65 years old of European ancestry from the Swedish SCAPIS cohort 2013–2018, for 1018 selected genetic variants previously with associated emphysema-related traits and lung function. SNPs associated in SCAPIS at a Bonferroni-corrected significant association (*p* < 4.9 × 10^–5^) are included in the table

Locus	Original study							Curremt Study (SCAPIS)			
	**Reference**	**SNP**	**Chr: Pos**	**EA**	**Pop**	**Trait**	**Beta**	**Model**	**Population**	**Beta**	**P**
Chr 2: *EFEMP1/MIR217HG/PNPT1*	3	rs62164518	2: 56,237,577	A	General	Lung function: FEV1/FVC	−6.9	binary	Ever smokers	0.40	3.2 × 10^–5^
Chr 4: *HHIP*	4	rs13140176	4: 145,489,098	A	General	COPD	0.17	ordinal	Total	0.17	3.5 × 10^–5^
								ordinal	Ever smokers	0.19	4.5 × 10^–5^
	7	rs13141641	4: 145,506,456	T	Ever smokers	Emphysema distribution: diff950	0.09	ordinal	Total	0.17	3.1 × 10^–5^
						Emphysema distribution: ratio950	0.12				
	8					% affected lung: %LAA-950	0.12				
						Emphysema level: Perc15	−2.2				
Chr 15: *CHRNA5-A3-B4*	7	rs9788721	15: 78,802,869	T	Ever smokers	Emphysema distribution: diff950	−0.08	binary	Total	−0.18	3.8 × 10^–5^
								binary	Current smokers	−0.40	1.4 × 10^–7^
						Emphysema distribution: ratio950	−0.09	binary	Ever smokers	−0.23	6.0 × 10^–6^
								ordinal	Total	−0.20	2.2 × 10^–6^
	6				Ever smokers	Having severe centrilobular emphysema	−0.01	ordinal	Current smokers	−0.40	1.6 × 10^–8^
								ordinal	Ever smokers	−0.24	4.6 × 10^–7^
	6	rs17486278	15: 78,867,482	C	Ever smokers	Having normal lungs	−0.03	binary	Current smokers	0.39	4.4 × 10^–7^
								binary	Ever smokers	0.23	7.3 × 10^–6^
	10				Ever smokers	DLCO	−0.12	ordinal	Total	0.19	6.6 × 10^–6^
								ordinal	Current smokers	0.39	4.8 × 10^–8^
								ordinal	Ever smokers	0.24	4.4 × 10^–7^
	7	rs12914385	15: 78,898,723	T	Ever smokers	Emphysema distribution: ratio950	0.11	binary	Current smokers	0.35	2.2 × 10^–6^
								binary	Ever smokers	0.21	3.1 × 10^–5^
								ordinal	Total	0.19	5.8 × 10^–6^
								ordinal	Current smokers	0.36	1.5 × 10^–7^
								ordinal	Ever smokers	0.22	1.4 × 10^–6^
	8	rs55676755	15: 78,898,932	G	Ever smokers	Proportion affected lung: %LAA-950	0.11	binary	Current smokers	0.41	8.7 × 10^–8^
								binary	Ever smokers	0.24	2.6 × 10^–6^
	4				General	COPD	0.10	ordinal	Total	0.20	2.3 × 10^–6^
								ordinal	Current smokers	0.41	7.0 × 10^–9^
								ordinal	Ever smokers	0.25	1.1 × 10^–7^
	10	rs112878080	15: 78,900,647	G	Ever smokers	DLCO	−0.13	binary	Current smokers	0.41	1.1 × 10^–7^
								binary	Ever smokers	0.24	2.9 × 10^–6^
								ordinal	Total	0.20	2.6 × 10^–6^
								ordinal	Current smokers	0.41	9.7 × 10^–9^
								ordinal	Ever smokers	0.25	1.4 × 10^–7^
	7	rs138544659	15: 78,900,701	T	Ever smokers	Emphysema distribution: diff950	−0.12	binary	Current smokers	−0.41	8.4 × 10^–8^
								binary	Ever smokers	−0.24	2.5 × 10^–6^
								ordinal	Total	−0.20	2.4 × 10^–6^
								ordinal	Current smokers	−0.41	7.7 × 10^–9^
								ordinal	Ever smokers	−0.25	1.2 × 10^–7^
	6	rs114205691	15: 78,901,113	C	Ever smokers	Having moderate centrilobular emphysema	−0.02	binary	Current smokers	−0.41	1.0 × 10^–7^
								binary	Ever smokers	−0.24	2.7 × 10^–6^
								ordinal	Total	−0.20	2.5 × 10^–6^
								ordinal	Current smokers	−0.41	8.9 × 10^–9^
								ordinal	Ever smokers	−0.25	1.2 × 10^–7^
	4	rs28534575	15: 78,923,845	T	General	COPD conditional on rs55676755)	0.10	ordinal	Total	0.20	3.2 × 10^–5^

*CHRNA5-A3-B4* nicotinic acetylcholine receptor cluster, *Chr* chromosome, *COPD* chronic obstructive pulmonary disease, *DLCO* diffusing capacity of the lungs for carbon monoxide, *Diff950* difference between emphysema percentage (%LAA-950) in the upper-third and lower-third of the lungs, *EA* effect allele, *HHIP* Hedgehog interacting protein, *Perc15* attenuation (HU) at the 15th percentile of the density histogram; Pop: study population, *Pos* genomic position (b37), *Ratio950* ratio of upper-third to lower-third emphysema, *SNP* single nucleotide polymorphism, *%LAA-950* emphysema percentage, i.e. percentage of CT densitometry low attenuation area less than − 950 Hounsfield Units (HU)

Eight of the 11 SNPs were in the previously well-known *CHRNA5-A3-B4* cluster and previously associated with COPD, DLCO, lobar distribution of emphysema (diff950 and ratio950), the proportion affected lung (%LAA-950) and normal lungs vs moderate and severe centrilobular emphysema (Table 3). In SCAPIS, these SNPs were associated at the replication Bonferroni level with emphysema in the total population and in the ever-smoker and current-smoker strata in both binary and ordinal models, but no associations were found in the never smoker and former smoker strata. For these eight SNPs, the effect was in the expected direction (increased risk of emphysema in SCAPIS) for alleles previously associated with the following phenotypes: higher ratio950 and diff950, higher risk of COPD, higher %LAA-950, higher risk of moderate and severe centrilobular emphysema, and lower risk of normal lungs.

The A-allele of the rs62164518 SNP on chromosome 2 in the *EFEMP1/MIR217HG/PNPT1* locus, previously linked to decreased FEV_1_/FVC [3] (Table 3), was associated with increased risk of emphysema in SCAPIS in the analysis of ever smokers.

Two variants at the *HHIP* (hedgehog interacting protein) locus on chromosome 4, previously reported positively associated with COPD, diff950 and ratio950, %LAA-950 and emphysema level (Perc15) were also associated with emphysema in SCAPIS in the expected direction in the total sample and in the ever-smoker analysis (Table 3).

## Discussion

We conducted a GWAS in the largest unselected general population sample studied for emphysema genetics to date. We identified three genetic loci significantly associated in smokers with emphysema scoring of CT images. Notably, we confirm the previously reported *CHRNA5-A3-B4* locus on chromosome 15, a well-known smoking and lung phenotype-related locus, alongside identifying two suggested novel associations on chromosome 2 and 3. We also assessed variants previously reported for emphysema and related traits, with Bonferroni-corrected testing, which suggested the previously reported association of rs62164518 on chromosome 2 (*EFEMP1/MIR217HG/PNPT1*) with FEV_1_/FVC ratio as an emphysema locus and supported the previously reported *CHRNA5-A3-B4* and *HHIP* findings as emphysema loci.

The strongest associations were found on chromosome 15 in the nicotinic acetylcholine receptor cluster (*CHRNA5-A3-B4*), repeatedly implicated in nicotine dependence, lung cancer, and COPD [4, 21, 22]. We found the association in both the current smoker and the “ever smoker” strata. This supports the hypothesis that smoking-related genetic variants contribute to emphysema risk, likely by increasing smoking intensity and cumulative tobacco exposure. However, the association persists even after adjusting for smoking, indicating additional direct biological effects or residual confounding. Studies show that *CHRNA5* and *CHRNA3* are expressed in the lung, and that a missense SNP in *CHRNA5* (rs16969968, Asp398Asn) is linked to lower *CHRNA5* mRNA levels. This may impair pulmonary repair capacity and airway integrity, increasing susceptibility to emphysema. Importantly, this effect has been observed even in never-smokers [23] and with bronchial hyperresponsiveness in children not exposed to cigarette smoke [24], suggesting that these genetic variants contribute to airflow obstruction independently of nicotine dependence. Additional research has found that silencing *CHRNA5* in bronchial epithelial cells disrupts cell adhesion and increases cell motility, which could further compromise lung repair mechanisms [25]. These findings clearly support the idea that *CHRNA5-A3-B4* locus variants influence emphysema risk through both smoking-related and direct effects on lung biology. Nonetheless, we could not find any significant association of *CHRNA5* locus with emphysema in the analysis restricted to never smokers.

Our proposed novel loci at chromosome 2 and 3 warrant external replication. Even after such replication, identifying the causal gene is not trivial. Of the genes in the loci, the candidate gene *DYSF* (dysferlin, chromosome 2) is of particular interest due to its established role in muscle membrane repair [26]. Traditionally associated with muscular dystrophy, dysferlin is a key calcium ion sensor involved in the Ca^2+^-triggered synaptic vesicle-plasma membrane fusion. A longitudinal study of 188 genetically confirmed dysferlinopathy patients revealed clinically significant respiratory impairment, with 24% of participants showing reduced FVC (< 80% predicted) at baseline, worsening to 30% by year 3 — even among ambulant individuals. This suggests that dysferlin deficiency directly impacts lung function, independent of skeletal muscle weakness [27]. Our top SNP (rs1365976) has previously not been linked to respiratory disease, however, another intron variant in DYSF (rs7566581) not in LD with our top SNP (D': 0.07; R^2^: 0.004) was previously associated with peak expiratory flow (*p* = 1 × 10^–17^) [3]. The other novel observed association rs13074352 at chromosome 3 lies in a broad LD block in an intergenic region, with the closest protein-coding gene being *ZBBX*. This variant has not been reported previously with any trait. However, about 300kB away within the same LD block, the variant rs10936486 (D': 0.47; R^2^: 0.07) has been associated with smoking initiation (p = 7 × 10^–10^) [28].

Our study expands on prior GWAS findings from COPDGene, ECLIPSE, and other studies, which examined CT-based quantitative emphysema traits in high-risk COPD populations. Our results from assessing prior genetic lung findings support previous *CHRNA5-A3-B4* and *HHIP* findings, and suggest rs62164518 in the *EFEMP1/MIR217HG/PNPT1*locus on chromosome 2 as an emphysema-linked variant. This variant was highlighted in the Shrine study because it showed heterogeneity across ancestries with largest effect size in Europeans and Admixture Americans [3].

Interestingly, our analyses revealed significant associations in current and ever smokers but not in never smokers. This aligns with previous research indicating that genetic contributions to emphysema risk may be amplified by environmental exposures such as smoking. However, our study design allowed us to detect potentially novel genetic determinants that operate beyond direct smoking-related pathways.

A major strength of this study is its large, unselected population-based sample, which reduces ascertainment bias compared to case–control studies focusing on severe COPD cases. Nevertheless, our study is novel in clearly demonstrating a lack of findings in a large sample of never smokers, suggesting that strong genetic signals are not present in this group. Additionally, our visual scoring approach captures emphysema in early stages, potentially allowing identification of genetic determinants of subclinical disease progression.

Limitations of this study should be considered when interpreting the findings. Visual scoring of emphysema, while standardized, is inherently subjective, and automated CT-based measures could provide more quantitative precision. On the other hand, a potential advantage of visual CT assessment is that it may be more sensitive to mild or focal emphysema that is not always captured by quantitative threshold-based measures. We used an ordinal score of summed lobar emphysema assessments to optimize power based on the available data. This score does not clearly differentiate between homogenous and localized emphysema of the same extent which might be a limitation; however, evidence regarding the potential importance of emphysema distribution is conflicting [29]. Our analysis was restricted to individuals of European ancestry to improve internal validity, which may limit generalizability to other populations; future studies in diverse ancestries are needed to explore genetic heterogeneity in emphysema susceptibility. While we adjusted for smoking status and pack-years, residual confounding by smoking intensity and potential exposure misclassification cannot be excluded. Although genetic risk factors would likely interact with environmental factors and disease pathways, we focused on potential main genetic effects, as the statistical power for gene-environment interactions would be low. The low prevalence of emphysema in never-smokers (*N* = 263, 2%) limited statistical power to detect associations. Our findings are based on a single cohort without independent replication and should therefore be interpreted with caution, given the possibility of false positive findings, as in other genetic association studies.

In summary, we identified three genome-wide significant loci for CT-scored emphysema, including one well-established smoking locus and two novel candidate loci. In never smokers, no signals were detected, suggesting lack of strong genetic drivers of emphysema in this group.

## Supplementary Information

Supplementary Material 1.

Supplementary Material 2.

Supplementary Material 3.

Supplementary Material 4.

Supplementary Material 5.

Supplementary Material 6.

Supplementary Material 7.

Supplementary Material 8.

Supplementary Material 9.

Supplementary Material 10.

## Data Availability

The data underlying this manuscript cannot be shared publicly for legal regulations related to the privacy of individuals that participated in the study. Data sharing requires ethical approval from the Swedish Ethics Review Authority and requests are made to the SCAPIS office.
