# Supplementary material for: Genome-wide association study of image-based emphysema scoring in the Swedish CArdioPulmonary bioImage Study (SCAPIS) suggests two new risk loci in smokers
Source: Respir Res. 2026 Aug 8;27:319. doi: 10.1186/s12931-026-03853-6 (PMC13501701; doi:10.1186/s12931-026-03853-6)
Supplement: Supplementary file 1 [file 12931_2026_3853_MOESM1_ESM.docx]

Supplementary Materials for:

**GWAS of emphysema based on visual scoring of CT images in 25639 participants from the SCAPIS cohort**

Fredrik Nyberg, Per Lundmark, Anders Blomberg, Arne Egesten, Jonas Eriksson Ström, Bruna Gigante, Anders Gummesson, Cecilia Gunnarsson, Christer Janson, Andrei Malinovschi, Anna-Carin Olin, Marju Orho-Melander, Hans Lennart Persson, Ida Pesonen, Magnus Sköld, Stefan Söderberg, Hanan Tanash, Lowie E.G.W. Vanfleteren, Tove Fall.

**Contents**

Page

Supplementary Methods	S2

Supplementary Fig. 1	S4

Supplementary Fig. 2	S5

**Supplementary Methods**

***CT imaging (additional details)***

A multidetector computed tomography (MDCT) scan of the chest was performed using a dedicated dual source CT scanner equipped with a Stellar Detector (Somatom Definition Flash, Siemens Healthineers, Forchheim, Germany). Scans were performed supine in full inspiration with a low dose protocol with the following parameters: Detector configuration (mm) 128 x 0.6, tube voltage (kVp) or reference level for Care (kV) 120, tube current (mAs) or reference dose for Care Dose 4D 25, rotation time 0.5 seconds, pitch 0.9, Matrix size 512 x 512. Image reconstruction parameters: slice thickness 0.6 mm, increment 0.6 mm, kernel B31f. No intravenous contrast was used. For radiation considerations, no repeat examinations were allowed. The median effective radiation dose was 2 mSv.

***Imputation of additional genotypes***

Out of the 30,154 individuals in the SCAPIS cohort, 29,433 individuals with blood samples and consent were submitted for DNA extraction at the biobank at Karolinska Institutet. All samples except two with low DNA concentration were successfully genotyped at NGI Uppsala, resulting in a dataset of 29,431 genotyped samples. This data was subjected to pre-imputation quality control where 4 samples were discarded due to sex genotype/phenotype discordance and 2 samples due to low call rate. The input dataset for imputation was generated by filtering samples (n=29,431) and SNPs based on concordance between phenotypic and genotypic sex, marker and individual call rates in two stages (≥90% initially, then ≥98%), Hardy-Weinberg equilibrium (HWE) testing at *p*≥1×10⁻⁸ in Swedish-origin samples, and a minor allele frequency (MAF) threshold of ≥0.1%. The data were then processed using Will Rayner’s imputation preparation script (HRC-1000Gcheck-bim-v4.3.0, [www.well.ox.ac.uk/~wrayner/tools](http://www.well.ox.ac.uk/~wrayner/tools)) to ensure consistency with the reference panel, checking for strand orientation, allele assignments, positions, reference/alternative allele concordance, and frequency differences. Palindromic SNPs with a MAF ≥40% were removed to minimize allele switch errors, along with SNPs that did not match the reference panel or had a >20% allele frequency difference between the dataset and reference panel. After excluding four samples due to sex genotype/phenotype discordance and two due to low call rates, these filtering steps resulted in an imputation input dataset of 29,425 samples and 538,960 markers. Genotype imputation was performed using the HRC r1.1 reference panel^13^ at the Sanger imputation service (imputation.sanger.ac.uk), employing the pre-phasing and imputation with EAGLE v2.4^29^ and PBWT pipeline.^30^ The HRC panel comprises 40.4 million variants, of which 7,906,639 remained after filtering for imputation quality (info score ≥0.7) and MAF ≥1%.

***Selection of final sample set used in the GWAS analysis***

To create the final GWAS genotype data the imputed dataset of 29,425 samples was filtered as follows: Samples with sex aneuploidy (n=11): markers were pruned for linkage disequilibrium with “plink –indep-pairwise 50 5 0.2“ and sex checks performed with “plink –check-sex ycount”. Samples were labeled as genotypic females if F<0.75 and Y count <500 and males if F>0.75 and Y count >500. If samples had female F-values and male Y counts they were called as XXY, and female Y count with male F-values were called as X0 and removed from the dataset. Non-European ancestry (n=2048): Subjects of non-European ancestry were excluded to avoid confounding by population stratification. Common variants between the study samples and the 1000 genomes^31^ were extracted and the study samples merged with the 1000 genomes dataset. Principal component analysis (PCA) was run on LD-pruned data with minor allele frequency ≥5% filtered for long-range high LD regions. Principal components were calculated in unrelated samples and then all samples were projected onto these principal components. Boundaries were set in PCs1, 3 and 4 to filter out samples with non-European origin according to reported parental birth country and comparisons to 1000 genomes reference samples.

Heterozygosity within 3SD of the mean (n=239): The raw heterozygosity was calculated on LD-pruned data as the proportion of non-missing genotypes that are heterozygous, based on output from “plink –het” (h=(Nnm-Nhom)/Nnm). An adjusted heterozygosity was then calculated by adjusting the raw heterozygosity for geographic origin by a quadratic model with interaction terms using the six first principal components in the 1000 genomes / SCAPIS PCA. The adjusted heterozygosity was then used for filtering purposes. Samples were removed if an adjusted heterozygosity fell outside 3 standard deviations from the mean.

Sample genotype call rate <99% (n=120). A strict sample call rate threshold was implemented due to the excellent performance of the samples in genotyping. This filtering resulted in a genotype dataset of 27,007 quality-controlled samples. For the analysis of the main outcomes in the current study, individuals with missing high-quality imaging (n=661) and missing data on smoking (n=707) were omitted, leaving 25,639 individuals for GWAS analysis (see flow chart in Supplementary Fig. 1).

**Supplementary Figures**

**Supplementary Fig. 1.** Flow chart describing the selection of the analysis cohort for this study (n=25639) from the Swedish SCAPIS cohort 2013-2018 of adults 50-65 years old.

CT = computed tomography [scan]

**Supplementary Fig. 2.** Quantile-quantile-plots for results in the main analyses for visual scoring of emphysema as a binary and ordinal variable, in adults 50-65 years old of European ancestry from the Swedish SCAPIS cohort 2013-2018 with GWAS, emphysema and smoking data available. A) Analysis in current smokers (n=3,161) for the binary outcome; B) analysis in ever smokers as a binary outcome; C) Current smokers (n=3,161), ordinal outcome; D) Ever smokers (n=12,619), ordinal outcome.
